# Cystoid macular edema associated with preservative-free latanoprost after uncomplicated cataract surgery: case report and review of the literature

**DOI:** 10.1186/s13104-017-2448-5

**Published:** 2017-03-20

**Authors:** Olga E. Makri, Foteini N. Tsapardoni, Panagiotis Plotas, Nikistratos Ifantis, Paraskevi T. Xanthopoulou, Constantine D. Georgakopoulos

**Affiliations:** 1 Department of Ophthalmology, Medical School, University of Patras, 265 04 Patras, Greece; 2 Department of Ophthalmology, 401 General Military Hospital of Athens, Athens, Greece

**Keywords:** Cystoid macular edema, Latanoprost, Preservative free, Uncomplicated phacoemulsification

## Abstract

**Background:**

Cystoid macular edema associated with latanoprost administration has been reported in patients after complicated cataract surgery with coexisting risk factors. We present the first case of preservative free latanoprost associated cystoid macular edema that occurred many months after uncomplicated cataract surgery.

**Case presentation:**

A 65-year old Caucasian female presented in the Outpatients Clinic complaining of reduced vision and metamorphopsia in the right eye. She had undergone uneventful phacoemulsification 19 months ago in the right eye and was under treatment with preservative free latanoprost eye drops for the last 7 months for ocular hypertension. Her remaining medical and ocular history were otherwise unremarkable. Cystoid macular edema with serous retinal detachment was diagnosed in the right eye using optical coherence tomography and fluorescein angiography. Latanoprost was discontinued and brinzolamide and nepafenac eye drops were administered in the right eye. Two months later, cystoid macular edema completely resolved with restoration of visual acuity. Nepafenac eye drops were administered for another 2 months. Eight months after latanoprost cessation optical coherence demonstrated no sign of cystoid macular edema whereas a subtle epiretinal membrane was noted.

**Conclusions:**

Cystoid macular edema may potentially occur in patients receiving preservative free latanoprost. More interestingly, in our case it was diagnosed in a patient with a long standing pseudophakia after uncomplicated phacoemulsification. No obvious risk factor for macular edema development was recognized. Prompt diagnosis and latanoprost discontinuation resulted in complete resolution of the cystoid macular edema and functional restoration of the eye.

## Background

Latanoprost (LP) is a phenyl-substituted prostaglandin analogue (PGA) that has become the most popular antiglaucoma medication for the treatment of primary open-angle glaucoma and ocular hypertension. Administered topically, LP lowers the intraocular pressure (IOP) presumably by increasing the uveoscleral outflow. The adverse effects of LP have been relatively mild and topical, mainly consisting of hypertrichosis, increased eyelash pigmentation, topical irritation, conjunctival hyperemia, superficial punctate keratopathy, and increased iris pigmentation while cystoid macular edema (CME) is considered a rare adverse effect [1].

There are several case reports presenting CME in early postoperative pseudophakias in eyes that are administered topical PGAs like LP, travoprost, bimatoprost, or unoprostone [2]. In the majority of these cases widely accepted risk factors that may potentially alter the blood–retinal barrier (BRB) and increase the risk of CME coexisted. These conditions include aphakia, complicated cataract surgery, vitreous loss, excessive intraoperative manipulations such as mechanical pupil stretch or iris prolapse during surgery, absent or ruptured posterior capsule, the presence of an anterior chamber intraocular lens, filtering or other glaucoma operations and intraocular surgeries, epiretinal membrane, history of uveitis or prior CME, retinal inflammatory or vascular disease like diabetes mellitus etc. [3, 4].

Latanoprost-related CME after uncomplicated phacoemulsification in absence of any systemic or ocular risk factors for developing CME is uncommon. There are reports of CME in six eyes treated with LP where the only recognized risk factor was previous uncomplicated cataract surgery [3]. However, details about these cases are lacking and, as the authors state, risk factors may be underestimated since five of them were spontaneous reports and one occurred during a phase III clinical trial. Yeh et al. report four cases where CME was clinically diagnosed 1 month after uneventful phacoemulsification, while LP was not discontinued perioperatively [4]. Thereafter, ten cases of PGA associated CME after uneventful cataract surgery in patients without known risk factors are described in more detail (Table 1) [5–11].

**Table 1:** Published cases of prostaglandin analogue related cystoid macular edema after uncomplicated cataract surgery in eyes without other risk factors

Study	PGA	Time from cataract surgery and CME diagnosis	Duration of PGAs treatment	Outcome	Action taken	Additional information
Costagliola et al. [5]	LP	2 years	5 days	Complete resolution of CME within days	LP discontinuation and treatment with diclofenac	
Altintas et al. [6]	LP	10 days	3 years	Complete resolution of CME in 3 months	Discontinuation of LP and initiation of topical and oral acetazolamide	Pseudoexfoliative glaucoma
						LP not discontinued perioperatively
	LP	1 month	2 months	CME resolved in 4 months	Initiation of ketorolac eye drops and oral acetazolamide	LP was discontinued 1 day preoperatively and was never re-administered
						Extracapsular extraction
Dhingra et al. [7]	LP	3 months	Unknown	CME resolved	LP discontinuation and sub-Tenon’s triamcinolone injection	Only clinical diagnosis
						LP not discontinued perioperatively
Ozdemir et al. [8]	LP	2 years	1 month	CME resolution in 3 weeks	LP discontinuation	Extracapsular cataract extraction
						Serous retinal detachment
Agange et al. [9]	LP	4 months	Years	CME resolved	LP discontinuation and treatment with diclofenac	Advanced pigmentary glaucoma
				CME recurred twice after PGA administration		LP was resumed immediately postoperatively
Panteleontidis et al. [10]	LP	3 months	4 years	Complete resolution of CME 1 month later Recurrence of CME after LP administration	Discontinuation of LP	Pseudoexfoliative glaucoma
						No statement of LP discontinuation perioperatively
	LP	3 weeks	7 years	Complete resolution of CME 3 weeks later	LP discontinuation and treatment with ketorolac	Pseudoexfoliative glaucoma
						No statement of LP discontinuation perioperatively
	LP	1 month	2 years	Complete resolution of CME 3 weeks later	LP discontinuation and treatment with ketorolac	Pseudoexfoliative glaucoma No statement of LP discontinuation perioperatively
Sacchi et al. [11]	Tafluprost	2 months	1 year	Refractory CME which resolved after an intravitreal implant of dexamethasone	Tafluprost discontinuation	Preservative free tafluprost
					Treatment with topical nepafenac and oral indomethacin, sub-Tenon’s, dexamethasone intravitreal implant	Serous retinal detachment
						LP not discontinued perioperatively

*PGA* Prostaglandin analogue, *CME* Cystoid macular edema, *LP* Latanoprost

In the following report we describe a patient with a history of uncomplicated phacoemulsification surgery 19 months ago who developed CME 7 months after initiation of preservative free LP.

## Case presentation

**Fig. 1:**
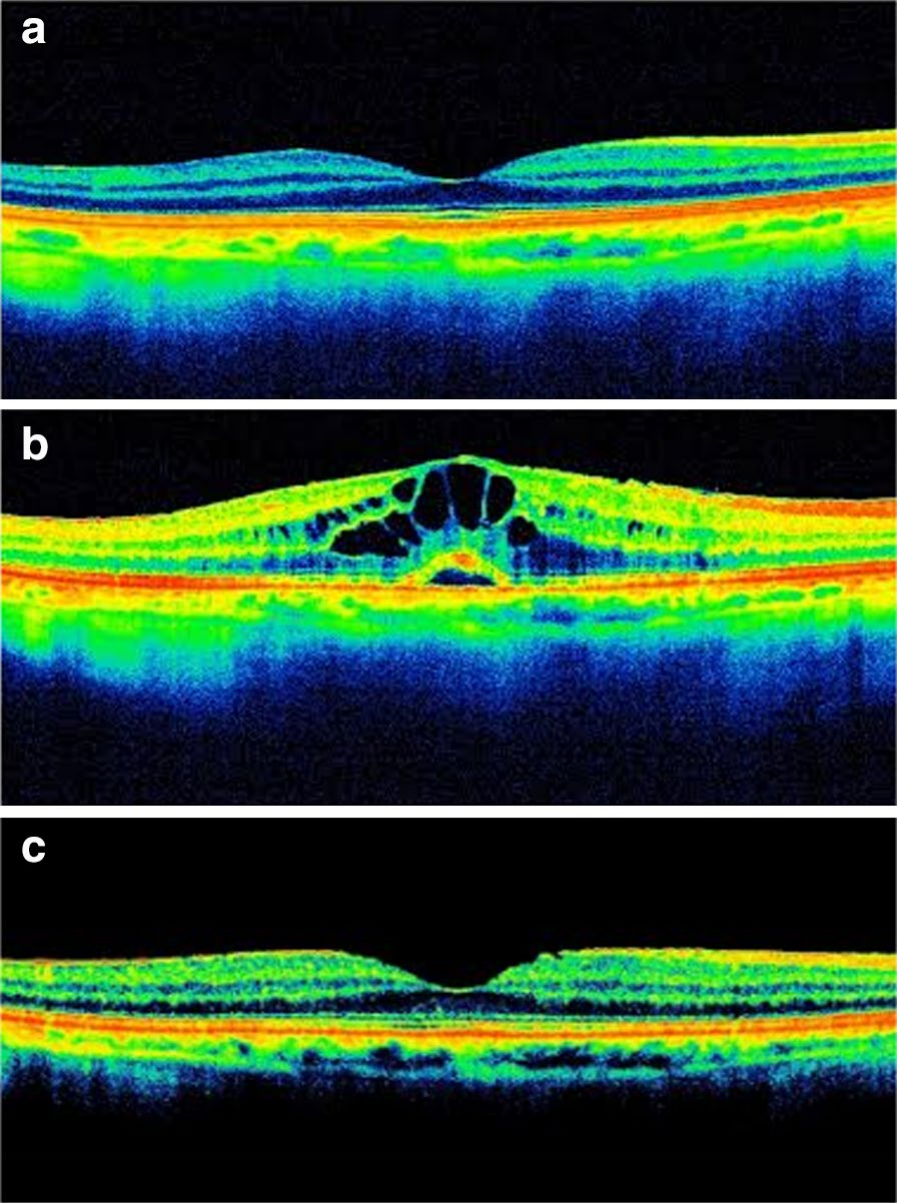
**a** Optical coherence tomography of right eye before latanoprost administration. No pathology is detected. **b** Seven months after treatment with preservative free latanoprost optical coherence tomography in right eye revealed cystoid macular edema with well-defined, intraretinal cystic areas of low reflectivity in the macula with serous retinal detachment. **c** Two months after latanoprost discontinuation optical coherence tomography demonstrated complete resolution of cystoid macular edema. A subtle epiretinal membrane is noted

A 65-year old Caucasian female presented in the Outpatients Clinic complaining of gradually reduced vision and metamorphopsia in the right eye (OD). Patient’s medical and ocular history were unremarkable. She had undergone uncomplicated cataract surgery with posterior chamber intraocular lens (IOL) implantation in OD 19 months ago and was on treatment with preservative free LP 0.005% (Monoprost 50 µg/ml, Thea, Clermont-Ferrand, France) every night in both eyes (OU) for the last 7 months due to ocular hypertension. At the time that LP ophthalmic solution was initiated patient’s best-corrected visual acuity (BCVA) was 20/20 OU and intraocular pressure (IOP) was 24 mm Hg OU while Optical Coherence Tomography (OCT), conducted as baseline examination, was normal (Fig. 1a).

**Fig. 2:**
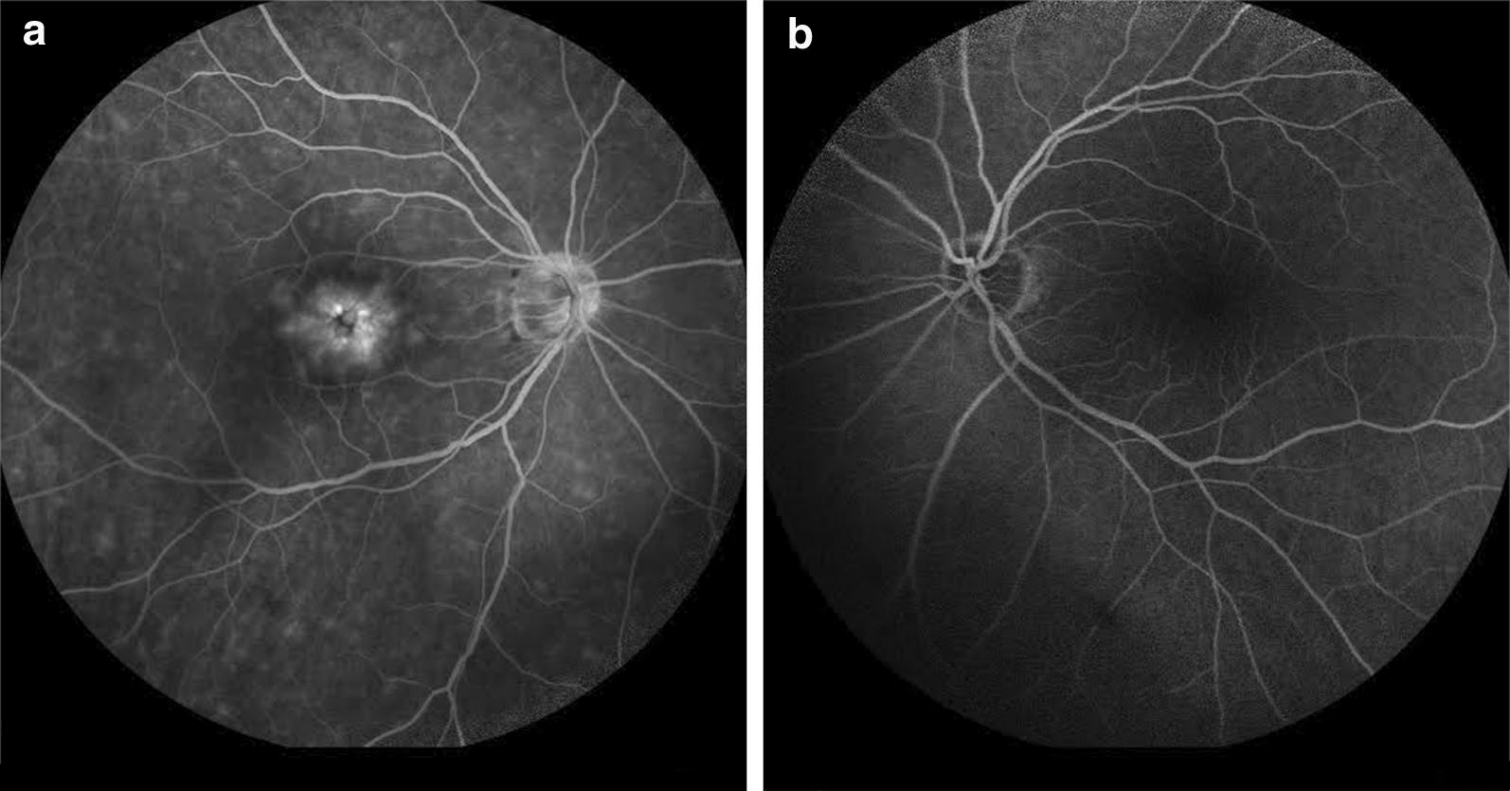
**a** Fluorescein angiography in the right eye revealed a petaloid leakage from the perifoveal retinal capillaries during the late phases without staining of the optic disc. Multiple areas of subtle fluorescein leakage were noted throughout the retina. **b** No leakage or any other pathology was observed in the left eye

On admission, ocular examination disclosed BCVA 20/30 in OD and 20/20 in OS. Slit lamp examination of OD revealed IOL in the capsular bag with intact posterior capsule with no evidence of intraocular inflammation or any other pathology such as ruptured posterior capsule. Slit lamp examination of OS was normal. Intraocular pressure was 14 mmHg OU. Fundus examination revealed a slight decrease in the foveal reflectivity OD indicative of CME whereas no pathology was observed in OS. Optical coherence tomography in OD confirmed the diagnosis of CME demonstrating well-defined, intraretinal cystic areas of low reflectivity in the macula, mainly in the outer plexiform and inner nuclear layers of the retina, with serous retinal detachment (SRD) (Fig. 1b). Fluorescein angiography (FA) in OD revealed a petaloid leakage from the perifoveal retinal capillaries during the late phases without staining of the optic disc, while multiple areas of subtle fluorescein leakage was noted throughout the retina (Fig. 2a). No leakage or any other pathology was observed in the OS (Fig. 2b).

Latanoprost was discontinued and was replaced with brinzolamide eye drops 1% b.i.d (Azopt 10 mg/ml, Alcon Laboratories Ltd., Hertfordshire, UK) OU. Nepafenac eye drops (Nevanac, 1 mg/ml, Alcon Laboratories Ltd., Hertfordshire, UK) t.i.d. were administered in OD. One month later, BCVA was restored to 20/20 in OD, metamorphopsia disappeared, and OCT demonstrated subsidence of CME with a single remaining intraretinal cyst. Two months after LP discontinuation, OCT demonstrated complete resolution of CME whereas a subtle epiretinal membrane was noted (Fig. 1c). At that time BCVA was 20/20 OU and IOP 17 mmHg in OD and 18 mmHg in OS. Nepafenac eye drops were administered for additional 2 months. Patient was examined every month thereafter. Eight months after LP cessation patient had BCVA 20/20 OU and IOP 18 mmHg OU, while she remained on brinzolamide eye drops b.i.d. OU. Optical coherence tomography demonstrated no sign of CME.

## Discussion

We describe the occurrence of CME with SRD in a pseudophakic patient 7 months after LP initiation and 19 months after uneventful phacoemulsification cataract extraction with posterior chamber IOL implant and intact posterior capsule. The diagnosis of CME was established with the characteristic OCT and FA findings, whereas no other possible causes of CME could be recognized in that eye. Although the patient was pseudophakic in OD for 19 months, there was no evidence of pseudophakic CME before LP initiation as it was indicated by clinical examination and a reference OCT, while CME was developed only in the pseudophakic eye treated with LP. Cystoid macular edema resolved with LP discontinuation and administration of nepafenac eye drops.

The exact mechanism by which LP may cause CME remains unclear. While LP does not seem to exhibit direct vasoactive or inflammatory properties, it is assumed that in early postoperative pseudophakias, LP affects the wound healing process of lens epithelial cells accelerating the biosynthesis of endogenous prostaglandins and other inflammatory mediators that eventually enhance disruption the blood-aqueous-barrier resulting in increase of incidence of angiographic CME [3, 12]. It is proposed that several risk factors such as complicated cataract surgery, vitreous loss, absent or ruptured posterior capsule render some eyes more prone to develop PG related CME [3, 4]. However, the vast majority of pseudophakic eyes treated with PGAs, even those with additional risk factors, do not develop CME [3]. Interestingly, our case does not have an obvious risk factor for developing CME except from the pseudophakic status itself.

Reviewing the cases in Table 1, four cases have a common feature which is pseudoexfoliative glaucoma [6, 10]. Pseudoexfoliation could be recognized as an independent risk factor since it results in abnormal blood-aqueous barrier predisposing to CME after phacoemulsification [13]. In two other cases CME occurred after uncomplicated extracapsular cataract extraction [6, 8]. In these cases someone could argue that the intraoperative manipulations during extracapsular cataract extraction could pose a risk factor that could interfere with the BRB integrity [14]. Of the remaining cases there are three cases of PGA associated CME where PGA had been already administered before surgery without being discontinued perioperatively and during the early postoperative period when the BRB is unstable [7, 9, 11].

In our case and in the case reported by Costagliola et al. [5] the CME occurred in eyes with long standing pseudophakias. In the case reported by Costagliola et al. [5] LP was prescribed 24-month after phacoemulsification and CME was diagnosed 5 days after initiation of LP treatment. Our patient had never received any antiglaucoma treatment until 12 months after surgery. While one could expect restoration of BRB in eyes with long standing pseudophakias, these two cases imply that blood-ocular barrier might remain fragile to LP several months after uncomplicated phacoemulsification even in eyes without other risk factors for BRB disruption.

Interestingly, it has been proposed that the main cause of CME in early postoperative pseudophakias is not LP, but rather the benzalkonium chloride, added as preservative in many antiglaucoma eye drops [15]. Our case is the first reported case of CME associated with preservative free LP and in combination with the case reported by Sacchi et al. weakens the theory of preservative-induced CME [11].

## Conclusions

We report a case of CME with SRD associated with LP administration with some interesting features. This is the first report of preservative free LP-induced CME. Furthermore, the CME occurred in an eye with a long standing pseudophakia after uncomplicated phacoemulsification. In our case no significant risk factor for CME development was recognized. Although the evidence for a causal association might be questioned and would be even more robust if CME recurred with rechallenge with LP, balancing the risk–benefit ratio we felt that it was unethical to rechallenge our patient in this setting because of the possibility of permanent visual loss [16]. The CME and SRD responded promptly to LP discontinuation and nepafenac administration. This temporal association suggests a causal relationship between topical application of LP and CME in our patient. Interestingly, the presence of pre-treatment normal OCT enhances a causal relationship.

There is not a single medication or surgical treatment without potential adverse effects and complications. Our case indicates the possibility of unexpected events related to LP administration after uncomplicated cataract surgery even in patients without any apparent risk factors.

## Abbreviations

BCVA: best-corrected visual acuity
BRB: blood–retinal barrier
CME: cystoid macular edema
FA: fluorescein angiography
IOL: intraocular lens
IOP: intraocular pressure
LP: latanoprost
OCT: optical coherence tomography
OD: right eye
OS: left eye
OU: both eyes
PGA: prostaglandine analogue
SRD: serous retinal detachment
